# Enhancing Observer Learning in Paediatric Resuscitation Simulations via Real-Time Telecommunication Tools

**DOI:** 10.7759/cureus.104835

**Published:** 2026-03-07

**Authors:** Devaki Visvalingam, Su Ann Khoo, Sashikumar Ganapathy, Gene Ong

**Affiliations:** 1 Paediatric Emergency Department, KK Women's and Children's Hospital, Singapore, SGP

**Keywords:** cognitive load, micro-debrief, observer learning, paediatric resuscitation simulation, real-time telecommunication tool

## Abstract

Objectives: This study aimed to enhance observer learning in high-fidelity paediatric resuscitation simulations by implementing real-time telecommunication tools, such as WhatsApp (Meta Platforms, Inc., Menlo Park, CA).

Methods: Paediatric resuscitation simulation workshops were conducted in 2023 and 2025, involving 41 participants. A structured observer programme utilised WhatsApp for dynamic interaction, enabling facilitators to engage observers with targeted questions and reinforce learning. Post-workshop surveys, using a five-point Likert scale and free text, evaluated the learning experience. The data were analysed quantitatively and qualitatively.

Results: Both workshops achieved a 100% survey response rate. Participants unanimously agreed or strongly agreed on administration quality and learning objective achievement. Qualitative data highlighted the benefits of micro-debriefs and WhatsApp for interaction and reinforcement. However, a key challenge was multitasking between observing the simulation and engaging with WhatsApp prompts, indicating potential cognitive overload.

Conclusion: Real-time telecommunication tools like WhatsApp can significantly enhance observer learning in paediatric resuscitation simulations. Careful timing and balancing digital interactions are critical to improving the educational experience and preventing cognitive overload.

## Introduction

The roles of simulation observers and their learning experience are often less well explored than those of active participants in simulation training [1]. Similarly, there is limited literature on teaching support structures that optimise roles and learning for both active participants and observers as well in simulation [2]. Simulation teaching must aim to provide equal and active learning opportunities for all who participate, whether directly involved as the performing team or as observers [3].

Assigning learners to roles that are purely supportive or focus on isolated tasks without full engagement in decision-making or broader team dynamics can lead to uneven learning benefits [4,5]. If the primary focus is rapid knowledge application in high-stakes scenarios, there is a risk of overlooking the development of crucial psychomotor skills or human factors, such as communication, teamwork, leadership, and situational awareness. All these elements are interconnected and crucial in real-world clinical practice [6,7]. Several learning theories and principles have been shown to enhance the effectiveness of simulation education programmes [3,8].

During simulation sessions, observers frequently engage in informal, parallel discussions about clinical decisions, evolving differential diagnoses, and the realism of presented findings, yet many do not voice those reflections during the formal debrief. This was an observation from our own simulation sessions that we had conducted in the past. Such silent observer behaviour, which may often be driven by hierarchy, uncertainty about debrief norms, or limited psychological safety, risks losing valuable learning opportunities. Studies have shown that learning outcomes for observers are improved through learner engagement and the use of observer tools [1]. As such, we wanted to use an observer tool to facilitate discussion, lower the barrier for participation for quiet learners and provide the facilitator with concrete material to deepen reflective discussion during debriefing.

We aimed to improve the learning experience for observers by implementing an educational programme during a high-fidelity paediatric resuscitation simulation workshop. We used learning theories, including (a) Experiential Learning Theory (Kolb’s reflective learning cycle), (b) Constructivism and Adult Learning Principles, (c) Social Cognitive Theory (SCT), and (d) Rapid Cycle Deliberate Practice (RCDP), to guide the structure of our observer learning [9-13]. We utilised a real-time communication platform to maintain observer engagement, facilitate learning through observation and discussion of others' actions, and deliver prompt, iterative feedback via RCDP micro-debriefs.

We hypothesised that the use of real-time telecommunications platforms, such as WhatsApp (Meta Platforms, Inc., Menlo Park, CA), for observers in a high-fidelity simulation training workshop will improve the learning experience by allowing dynamic interaction between observers and facilitators to provide opportunities for observers to ask questions during the scenario development, allow facilitators to engage observers by asking targeted questions during critical scenario development without affecting the simulation development, and actively listen and gather observers’ comments and input to further explore during the debriefing session. Our goal was to assess the learner receptiveness towards this innovative method within a simulation education programme.

## Materials and methods

In 2023 and 2025, we organised paediatric resuscitation simulation workshops for advanced learners. This workshop was held during the Society for Emergency Medicine (Singapore) Annual Scientific Meeting, which is a biannual event. Participants were self-selected attendees for the workshop, and they ranged from residents to senior consultants, nurses, paramedics, and students. The main objective was to have hands-on practice and interactive group learning for the management of critically ill and injured paediatric patients through high-fidelity simulation training. To address the identified gaps in observer engagement and learning, we enhanced our existing workshop by implementing a structured programme specifically for observers. The structure was integrated into this four-hour paediatric resuscitation training session, ensuring that observers received targeted support and active learning opportunities throughout.

David Kolb's (1984) Experiential Learning Theory (ELT) posits that learning is a cycle where knowledge is created through concrete experience to reflecting, conceptualising, and then testing new ideas, making it learner-centric and vital for development [14]. This underpins our use of real patient care scenarios, followed by immediate reflection. Constructivism, a psychology of learning theory, is based on the work of Piaget and Vygotsky [10,15]. Constructivism theory holds that assimilation, accommodation, and construction are the basic operating processes in learning [15]. Our workshop engaged the observers actively, via a real-time communication platform, to help them reflect and construct understanding during the simulation scenario. Bandura's SCT explains learning as a dynamic, reciprocal interaction between personal factors, behaviours, and the environment, emphasising observational learning, where people learn by watching others [11,16]. RCDP is a simulation-based training approach widely used in medical education. It divides difficult skills into smaller, easier-to-handle parts and gives quick, regular feedback (micro-debriefs) during scenarios [12]. As this was being done during the simulation session with the active participants, observers also learnt from this approach, and there were also prompts given through the telecommunication platform to clarify any misconceptions and enhance their learning at the same time. With these learning theories as our foundation, we sought to maintain the engagement of our observers while also providing direction for their thought processes.

Design of the workshop

Four paediatric resuscitation scenarios were developed. The scenarios included acute management of critically ill and injured infants and children (in both medical and trauma aspects). To enhance the observer learning, we incorporated two modalities: (1) WhatsApp as the telecommunication platform for the structured learning for the observers and (2) instruction and clinical summary sheets to allow observers to fill in the learning points for their future reference.

Participants were divided into groups of six to seven people. In each scenario, one group was the active performing team, while the other three groups were observers. Among the observers, one group was pre-assigned to lead the debrief discussion, and two groups participated in the WhatsApp polling and discussion. Each scenario ran for 30 minutes, followed by a 10-minute summary and rapid debrief. A WhatsApp group was formed with all the participants to engage the observers in discussion. WhatsApp was selected over other digital platforms for several reasons: it is widely used among participants, eliminating the need to download a new application; it is free of charge; and it offers a polling function that effectively facilitates group discussion. As participants were regular WhatsApp users in routine communication, no formal app training was given. The intervention required only basic functions such as reading, short text posts, and reacting to polls. A facilitator provided a brief orientation and real-time troubleshooting during the first scenario to support participation.

**Figure 1:**
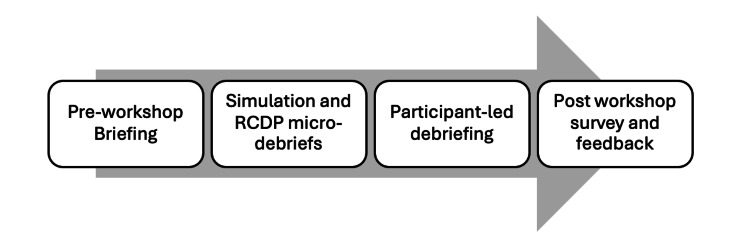
A pre-workshop briefing was done for introduction and group assignment, followed by simulation with micro-debriefs, participant-led debrief, and lastly post-workshop survey and feedback.

Each group took turns being active participants, being an observer to lead debriefs or engaging in WhatsApp discussions and polls for each scenario. One facilitator managed the simulation scenario with the performing team, while another facilitator concurrently engaged the observers through WhatsApp, posting questions and polls. A set of guiding questions was prepared for each scenario to prompt observer thinking at critical junctures that required decision-making; these were posted in the WhatsApp group in real time as the scenario progressed (Figure 1).

An example of a simulation scenario was a case of a five-year-old boy who presented with tachycardia, noted to be stable ventricular tachycardia, who became hypotensive. Questions that were posed to facilitate the discussion on WhatsApp while the simulation was ongoing were as follows: thoughts about the initial resuscitation and when to call for help; polling on the possible next line of action; priorities of management; how to prepare for defibrillation; whether a bolus should be considered for the hypotension; thoughts about starting inotropes early; and what shockable rhythms are.

Paper-based surveys were administered immediately after the workshop to assess the participants’ learning experience. We collected both quantitative and qualitative feedback. Participants rated statements about the workshop’s administration (orientation, scenario realism, mannikins, facilities) and the learning experience (knowledge gains, influence on future practice, environment, confidence, etc.) on a five-point Likert scale, along with an open text field for reflections and feedback.

We analysed the post-workshop Likert scale responses descriptively. Frequencies and percentages of agreement levels were calculated for each response option. We conducted an inductive thematic analysis of the free-text responses. Two authors independently reviewed the qualitative comments to identify recurring themes, resolving any differences in coding by discussion.

Ethical considerations

The research work complied with the Declaration of Helsinki. Studies involving the evaluation of courses and workshops are exempted from the Human Biomedical Research Act (HBRA) 2015, Singapore. Non-HBRA studies are exempt from oversight of human subject research by the institutional review board (IRB). Verbal and written consent was obtained from all participants for confidentiality for individual performances, the identities of participants/instructors, and all details specific to the course, as well as the use of any images or videos captured during the workshop and the anonymity of survey responses.

## Results

Both workshops achieved a 100% response rate on their post-workshop surveys (N = 24 for 2023; N = 17 for 2025). The dominant learner group shifted between years. In 2023, residents constituted the largest demographic (58.33%). In 2025, consultants formed the largest group (35.29%), followed by nursing personnel (23.53%) (Table 1).

**Table 1:** A total of 41 participants were involved in the two workshops held in 2023 and 2025. Combined learner profile (N = 41).

Designation	Combined Number (N = 41)	Year 2023	Year 2025
Residents	17	14	3
Consultants	10	4	6
Senior consultants	4	2	2
Associate consultants	4	3	1
Nursing	4	0	4
Paramedic	1	1	0
Student	1	0	1

Summarised quantitative findings

Both workshops achieved 100% agreement or strong agreement regarding the quality of administration (Table 2) and the achievement of learning objectives (Table 3). Notably, a larger share of participants in 2025 selected "Strongly Agree" on these items (88% in 2025 vs. 67% in 2023) for learning experience, suggesting an even more enthusiastic reception in the second iteration (Table 3).

**Table 2:** Evaluation of workshop/course administration (aspects covered: orientation, scenarios, mannikins, and room facilities).

Likert scale category	Year 2023 (N = 24)	Year 2025 (N = 17)
1 & 2 (Disagree)	0 (0%)	0 (0%)
3 (Neither)	1 (4.17%)	0 (0%)
4 (Agree)	12 (50%)	2 (12%)
5 (Strongly agree)	11 (45.83%)	15 (88%)

**Table 3:** Evaluation of paediatric emergency learning experience and debrief (aspects covered: bridging knowledge gaps, influence in future management, safe learning environment, improving confidence, micro-debriefs, and active participation).

Likert scale category	Year 2023 (N = 24)	Year 2025 (N = 17)
1 & 2 (Disagree)	0 (0%)	0 (0%)
3 (Neither)	0 (0%)	0 (0%)
4 (Agree)	8 (33.33%)	2 (12%)
5 (Strongly agree)	16 (66.67%)	15 (88%)

Summarised qualitative reflections

Free-text feedback predominantly described positive reactions to the interactive design, although participants also reported challenges. Key themes and representative quotes are summarised in Table 4. In both 2023 and 2025, participants noted that micro-debriefs were new to the majority but were highly beneficial in helping set the direction of the resuscitation and in decision-making. Participants consistently appreciated the real-time engagement, questions, and summary of learning provided via WhatsApp. This approach successfully facilitated enhanced interaction without interrupting the performing team. Emphasis on feedback from observers was noted to be beneficial, contributing to an interactive, multi-modal discussion.

**Table 4:** The qualitative feedback reflects common themes across both workshops regarding reflections and improvements.

Combined qualitative theme	Feedback quotes
Reflections	“The way it is done in a hybrid manner allowing everyone to participate and ask questions and comments in WhatsApp was beneficial"
	“Interactive, good level of complexity and multi-modal discussion was good”
	“Good real-life scenarios, mannikin quality, excellent briefs and de-briefs”
	“WhatsApp group feedback and participation is a novel way of engaging observers”
Improvements	“WhatsApp engagement does distract a bit from following the progress of the scenarios. To find a fine balance between watching and discussing.”
	“Sound quality was not great at times”
	“Allocate more time for the scenarios and the debrief sessions”
	“For provision of pre-course material for pre-reading”
	“Smaller group of participants would have been better"

The most critical challenge noted across both workshops was related to managing the interactive technology during the simulation. A core, recurring point of improvement was the difficulty of multitasking between WhatsApp polling questions and observing the scenario unfolding at the same time. Participants in both workshops requested a longer duration for scenarios and debriefs, or suggested that more time be allocated for debrief. Additionally, they suggested finding a better balance between engaging the WhatsApp group and observing the ongoing simulation. Additional improvements requested included provision of pre-course material, smaller groups of participants, and improved audio quality during the scenario (Table 4).

## Discussion

Our results demonstrate that incorporating a real-time communication tool, such as WhatsApp, for observers in simulation can significantly enhance their engagement and perceived learning. All participants responded positively to the approach, highlighting the value of features like micro-debriefs and digital question and answer (Q&A) to reinforce learning in real time. This aligns well with the established benefits of simulation-based education in improving both technical and non-technical skills in a safe environment [17,18]. These competencies will lead to faster, better decisions, reduced errors, and improved patient outcomes, like survival rates and shorter hospital stays, by bridging gaps from traditional learning to handling high-stakes paediatric emergencies effectively [19,20].

The challenge of traditional observer roles is the uneven learning opportunities between the active and passive participants [4,5,21]. As educators, we need to ensure the learning is equally beneficial to active participants as well as observers during simulation training. Active learning requires learners to engage actively with material through tasks like discussion, problem-solving, or hands-on practice, promoting deeper processing, better retention, and transfer of skills, whereas passive learning involves primarily receiving information from an instructor or demonstration with limited interaction [22,23].

Real-time telecommunication platforms, such as WhatsApp, can serve as a bridge to engage and enhance observer learning during ongoing simulation discussions. Instead of passively watching and receiving information, participants who become observers actively construct their understanding by posing questions, engaging in discussions, and synthesising observations in real time. This active processing, mediated by the digital platform, moves observers beyond mere spectatorship to become co-creators of knowledge within the simulation environment, becoming active learners [24].

In this workshop, there was also a deliberate rotation of roles: three groups included active participants, active observers who evaluate and provide feedback, and lastly, the group of observers who participated in the WhatsApp discussion. Bandura's social cognitive theory emphasises observational learning and modelling [11]. By allowing observers to interact, ask questions, and receive feedback via WhatsApp without interrupting the performing team, the platform significantly enriches the learning experience. Observers are not just being passive learners; they are required to actively observe and process the decisions and actions of the performing team while considering alternative perspectives through the digital discussion. All these can enhance their curiosity for future performance in similar clinical scenarios [25].

Evidence suggests that micro-debriefs, brief targeted discussions during or immediately after critical junctures, are highly effective in providing timely feedback, correcting misunderstandings, and solidifying learning points [5,26]. This immediate reflection is particularly potent in high-stakes environments, like paediatric resuscitation. This integration of micro debriefs with digital platforms accelerates reflection and conceptualisation in accordance with Kolb’s experiential learning cycle [9]. For observers, the "concrete experience" is observing the simulation. WhatsApp directly improves the "reflective observation" stage. By allowing observers to ask questions and share observations in real-time via the group chat, it provides a structured mechanism for immediate, collective reflection on events as they unfold. This immediate processing, facilitated by instructors or peers within the chat, can lead more rapidly to "abstract conceptualisation", forming new insights and understandings, than waiting for a full post-scenario debriefing [5,9].

Despite the numerous benefits, feedback indicates that multitasking between the WhatsApp discussion and observing the unfolding scenario presents challenges. This highlights potential issues of cognitive load [27]. While digital tools can enhance engagement, overstimulation or the need to divide attention between the live simulation and a digital interface can detract from deep processing and learning [28].

Future iterations must focus on strategic integration rather than mere addition. This could involve careful timing of WhatsApp prompts, clearer instructions on expected digital participation, or adapting the design of polling questions to require minimal cognitive effort during critical simulation phases. The goal should be to leverage the unique advantages of real-time communication without overburdening the observer's attention capacity and optimising their learning environment.

Our design of the workshop is a novel approach to integrate a telecommunication tool, such as WhatsApp, into simulation education. There has been recognition that instant messaging applications can enhance medical education by establishing communication pathways between students and tutors, creating learning opportunities, and ultimately offering a more enriched educational experience [29]. Telecommunication tools can transform observers from passive viewers into active, reflective participants, ensuring more equitable learning opportunities across all trainees.

The workshop structure effectively engages a larger number of observers, creating opportunities for scalability in simulation training, especially in situations where direct hands-on participation is limited. This is particularly relevant given the varied learner profiles, from "Senior Consultants" to "Students" and "Paramedics" across both workshops.

Limitations of the study

This study has several limitations that should be considered when interpreting its findings. First, the evaluation relied primarily on self‑reported participant feedback collected through post‑workshop surveys. Although subjective perceptions of learning, engagement, and satisfaction are important outcomes in educational research, they do not directly measure objective knowledge acquisition, skill performance, or transfer of learning to clinical practice. The findings, therefore, reflect perceived educational value rather than demonstrable improvements in competence or patient-related outcomes.

Second, the sample size was relatively small (41 participants across two workshops). Although the workshops included a heterogeneous group of learners (residents, consultants, nurses, paramedics, and a student), the limited number of participants and single‑country conference setting may restrict the generalisability of the findings to other contexts, learner populations, or healthcare systems.

Third, this study employed a descriptive, non‑comparative design without a control group. Without comparison to a traditional observer model or an alternative engagement strategy, it is not possible to definitively attribute improvements in observer engagement solely to the use of WhatsApp or to determine its relative effectiveness compared with other educational approaches.

Lastly, we did not measure baseline WhatsApp proficiency; therefore, we cannot exclude that differing familiarity influenced engagement. Given that the tasks required only basic messaging skills and on-site support was provided, we believe any effect of digital literacy on outcomes was small; future work should formally assess digital proficiency and consider training or alternative platforms for less-experienced users.

Despite these limitations, this study provides valuable preliminary insights into the potential role of real‑time telecommunication tools in enhancing observer engagement during simulation‑based education. Further research is needed to quantitatively measure the impact of such digital integration on observer learning outcomes, knowledge retention, and transfer to clinical practice. A more robust qualitative exploration is also required to identify enablers and factors within the context of the learning environment that can effectively support deeper and longitudinal learning within teams. Investigating different modalities of digital interaction, optimal timing for interventions, and instructor roles in facilitating digital discussions will be crucial for refining this promising pedagogical approach.

## Conclusions

The overall study findings indicate that the interactive use of the real-time telecommunication tool, WhatsApp, may be beneficial and can be used to enhance observer learning during simulation training. However, facilitators must be aware of the cognitive load induced by the addition of digital communication among observers and possible distractions that could arise from the tool. Optimising the timing and balance of this interaction remains the primary area for future improvement and objective evaluation. The purposeful creation of opportunities for equal and active participation among those performing and observing was pivotal to the overall enhancement of their learning experiences.
